# Factors influencing clinical symptoms and treatment of patients with traffic accident injuries: A retrospective chart review with a questionnaire survey

**DOI:** 10.1371/journal.pone.0252922

**Published:** 2021-06-18

**Authors:** Kwanghwi Kim, Ohhoon Kwon, Taeyeon Kim, Taegeol Lee, Kihoon Choi, Hyun Woo Cho, Doori Kim, Kyoung Sun Park, Jinho Lee, Byung-Cheul Shin, In-Hyuk Ha

**Affiliations:** 1 Haeundae Jaseng Hospital of Korean Medicine, Haeundae-gu, Busan, Republic of Korea; 2 Department of Korean Medicine, Graduate School, Kyung Hee University, Seoul, Republic of Korea; 3 Jaseng Hospital of Korean Medicine, Gangnam-gu, Seoul, Republic of Korea; 4 School of Korean Medicine, Pusan National University, Yangsan, Kyungnam, Republic of Korea; 5 Spine & Joint Center, Pusan National University Korean Medicine Hospital, Yangsan, Kyungnam, Republic of Korea; 6 Jaseng Spine and Joint Research Institute, Jaseng Medical Foundations, Gangnam-gu, Seoul, Republic of Korea; Tsinghua University, CHINA

## Abstract

This study analyzed factors influencing clinical symptoms and treatment of patients with traffic accident injuries. It used a retrospective chart review and questionnaire survey obtained from 560 patients (266 men and 294 women). It also conducted follow-up observations of progress after car insurance settlements and investigated the usefulness of and patient satisfaction with integrative Korean medicine treatment for traffic accident injuries. Retrospective data of patients admitted for traffic accident injury were obtained. A questionnaire survey was conducted to collect data regarding the degree of traffic accident damage, severity of pain at settlement, any treatment after settlement and duration and cost of such treatment, and patient satisfaction with car insurance services and Korean medicine treatment for traffic accident injury. The results showed no significant association between pain and the degree of damage to the car at the time of traffic accident (P = 0.662), although the degree of damage to the car was more significantly associated with time to reach a car insurance settlement than severity of pain in the patient (P = 0.003). There was no significant association between the degree of damage to the car in a traffic accident and pain after a traffic accident. Greater severity of pain at the time of the car insurance settlement was associated with greater cost and longer time spent in treatment after the car insurance settlement.

## Introduction

Traffic accidents (TAs) are a major cause of death and other disabilities in modern societies [1]. Annually, approximately 50 million people around the world suffer a serious injury from a TA [2]. In 2018, there were 1,228,129 TAs in South Korea, which involved injuries to 1,935,008 people. Property damage, casualties, and cost associated with TAs were estimated to be approximately $21 billion in 2018, which is equivalent to approximately 1.4% of South Korea’s gross domestic product [3]. Moreover, the recovery period for patients who suffer whiplash from a TA could be longer than expected, and 50% of individuals may exhibit symptoms for more than a year after the accident [4, 5]. Studies have shown that many people experience difficulties in returning to work or at work due to fears about moving around or over-sensitivity to pain after a TA [6].

Musculoskeletal disorders are often caused by TA-related trauma [7]. Neck and low-back pain are major symptoms that typically appear after a TA. A previous study reported that 36% of patients have pain that lasts less than a month, whereas another 36% have neck pain that lasts over a year [8]. Another study reported that it takes an average of 505 days to recover from low-back pain [9].

Whiplash is defined as the acceleration–deceleration mechanism of energy transfer to the neck [10]. Many countries have policies to address whiplash-related medical issues, and various ongoing studies examine the onset of whiplash and healthcare service utilization. Whiplash could cause damage to bone or soft tissues, which could exhibit a variety of clinical symptoms, called whiplash-associated disorders (WADs) [10]. Despite the increasing number of patients with a disability caused by whiplash, there is little epidemiological information and a scarcity of diagnostic and prognostic information, although there is a broad interpretation with respect to treatment [11]. Moreover, various studies have identified that WADs could lead to a high percentage of cases with chronic pain and disability. Despite this, WADs result in disagreements among patients, physicians, insurance companies, and government agencies [12].

South Korea has a dualistic healthcare system, consisting of both Western medicine and Korean medicine (KM); in particular, KM treatment is being applied to various musculoskeletal disorders, including pain caused by TAs. KM considers pain due to TAs to be caused by the obstruction and stagnation of Qi-blood and applies various treatment modalities, including herbal therapy, acupuncture, cupping, and Chuna therapy, to facilitate Qi-blood circulation [13]. KM treatment is gaining application worldwide, and because its efficacy has been identified to be more significant than that of analgesics for musculoskeletal disorders [14], it is increasingly suggested as a treatment [15–17].

In many TA cases, patients may complain about severe pain and functional impairment; however, diagnostic tests may not reveal the exact cause of pain. Consequently, patients with WADs are sometimes considered to be malingering, lying, or being neurotic [12]. A study by Nicholas et al. reported that there was no association between the direction of auto collision, degree of injury, and pain [18], but the general perception among people was that the severity of pain would be weak in cases involving a mild collision or no abnormal findings on diagnostic tests. These reasons alone could limit the treatment options, but not receiving proper treatment from a timely settlement could lead to various risks, including the pain becoming chronic, which could also limit patient satisfaction in treatment.

The present study examined retrospective data of patients, questionnaire surveys on accident details, and patient awareness about car insurance (CI) to analyze the following: How is the degree of TA damage associated with the severity of pain? What is the level of improvement in patients associated with the CI settlement? What are the factors associated with accident settlement? What is the treatment course of patients after settlement? Additionally, this study investigated patients’ perceptions about the current CI system and satisfaction in and significance of KM treatment for WADs. The findings of this study could be helpful in establishing and providing basic information needed for treatment coverage and healthcare practice guidelines about TA injury for relevant healthcare service providers, researchers, and policymakers.

## Methods

### Selection of the study population

#### Inclusion criteria

We included patients in the population who

1. had received inpatient treatment for a TA injury at a KM hospital between January 1, 2016, and December 31, 2018;
2. had reached a CI settlement for an existing TA case; and
3. were aged 19–65 years.

#### Exclusion criteria

We excluded patients from the population who

1. had a TA injury for which a CI settlement had not yet been reached;
2. had reached a CI settlement less than 90 days earlier; or
3. were unable to communicate.

### Ethics statement

This study was registered at www.clinicaltrials.gov (NCT04167930) and approved by the Institutional Review Board (IRB) of Jaseng Hospital of Korean Medicine (IRB approval no.: JASENG 2019-07-006) and was conducted in compliance with all relevant ethical guidelines. In addition, patient consent for the use of their personal information for academic purposes was obtained through a Google questionnaire survey (using Google Forms).

### Data collection and statistical analysis

Retrospective data were obtained using medical records and computer data on CI settlements of patients with TA injuries who were admitted to KM hospitals, and a questionnaire survey was conducted based on that data. The two sets of data were used to analyze the factors influencing the relationships. Further, electronic medical records were used to analyze the condition of the patients during their hospitalization, and a questionnaire was used to identify the situation after the CI settlement. Furthermore, patient memos and questionnaire responses were used to compare the symptoms according to the degree of TA damage.

### Retrospective data surveyed

Retrospective data were used for the objective assessment of patient conditions at the time of admission and discharge. An assessment of the quality of life (QoL) and neck and low-back pain experienced by patients with TA injuries was needed. Accordingly, the following items were surveyed. Each item was measured twice, once at admission and once at discharge.

#### Neck Disability Index (NDI)

This index is designed to assess the degree of neck disability in the daily life of a patient, and it uses a method that derives a mean score by dividing the total score by the number of questions answered [19]. It is constructed as a questionnaire with 10 items, and each item is graded on a scale of 0~–5 points for a total of 50 points.

#### Oswestry Disability Index (ODI)

This index is a questionnaire with 10 items, developed to assess the degree of disability associated with low-back pain [20]. Each item is divided into six stages and is graded on a scale of 0–5 points. Higher scores indicate a higher degree of disability. The present study used the official Korean version of ODI.

#### 5-level EuroQol 5-Dimension (EQ-5D-5L)

This tool measures health-related QoL (HR-QoL), which is widely used in the healthcare field [21]. EQ-5D-5L comprises five items on current health status (mobility, self-care, usual activities, pain/discomfort, and anxiety/depression), and each is graded across five stages (1: no problem, 2: slight problem, 3: moderate problem, 4: severe problem, and 5: extreme problem). In the present study, the weighted value for HR-QoL was calculated by applying the weighting model estimated for Koreans.

### Questionnaire items

For people who had reached a CI settlement, symptoms at the time of and after the CI settlement were investigated. The correlation between the CI settlement and alleviation of symptoms was investigated through a questionnaire that consisted of five major parts: demographic characteristics (Part 1), previous history of a TA injury and degree of TA damage (Part 2), severity of pain at the time of the CI settlement, treatment after the CI settlement, situation after the CI settlement (Part 3), satisfaction in symptom improvement at the time of CI settlement and reasons for settlement (Part 4), and satisfaction in KM treatment by patients with a TA injury (Part 5).

The questionnaire was developed based on the Google Forms platform [22]. The items in the questionnaire were used after development through several rounds of agreement by experts from the Jaseng Spine and Joint Research Institute, the Association of Korean Medicine, and the Department of Rehabilitation Medicine of Korean Medicine at Pusan National University Korean Medicine Hospital.

### Data processing of surveyed items

Age was classified into groups of <30, 30–39, 40–49, 50–59, and ≥60 years. Job was classified as engineer, office job, self-employment, service/sales, research, specialized, housewife, student, and others. Income was classified as <2, 2–4, and ≥4 million won monthly. WAD grade was originally classified into grades 0–4. However, the number of patients with WAD grade 4 was very low (only three). Accordingly, WAD grades 3 and 4 were grouped together in the present study. For the degree of TA damage, the response to the question, “What is the degree of damage to the car you were riding in at the time of the accident?” was classified as follows: Grade 1 –no damage; Grade 2 –scratches; Grade 3 –partially crushed; Grade 4 –seriously crushed; Grade 5 –broken in half; and Grade 6 –scrap. Pain at the time of and after the accident was classified on the numeric rating scale (NRS) of 0–3, 4–6, and ≥7 points. Duration to reach the CI settlement was classified into <1 week, 1–4 weeks, 4 weeks–3 months, 3–6 months, and ≥6 months, and mean ± standard deviation (SD) was expressed in days. Demographic characteristics, severity of pain at the time of accident and CI settlement, duration to reach the CI settlement, degree of the TA damage, and WAD grade were assessed by sex.

### Statistical analysis

Continuous variable data were expressed as mean and SD. Categorical variables were expressed as numbers and percentages. Pain and disability-related outcomes by TA grade were also expressed as mean and SD. Outcomes between TA grades were compared through analysis of variance (ANOVA), and the P-value was presented.

Duration to reach CI settlement by TA grade, NRS for pain after TA, and WAD grade were expressed as mean and SD. The duration to reach CI settlement between TA grades, NRS for pain after TA, and WAD grade were compared through ANOVA, and each P-value was presented.

Medical expenditure and treatment duration after CI settlement by NRS for pain after CI settlement and basic characteristics were expressed. For each characteristic, differences in expenditure and treatment duration were tested using ANOVA, and the P-value was presented.

The linear regression model was used to analyze the change in medical expenditure and treatment duration according to NRS for pain after CI settlement. Medical cost was log transformed by adding 1 to all values. Model 1 included pain at the time of the CI settlement; Model 2 included Model 1, age, and sex; and Model 3 included Model 2, income, and job. R-square and P-values were given for each model.

## Results

### Study flow

For this study, 3,005 patients who gave their consent for the use of their personal information and to receive future SMS notifications were selected. Data were collected by sending information about participating in the study and the survey address to the selected patients during October 1–15, 2019. Data from 619 of the 680 patients who responded were obtained after excluding those with missing data.

These data were used to investigate the overall satisfaction level and costs. The association between the degree of TA damage and patient condition was analyzed for 506 patients in the in-car TA injury group. Patients with out-of-car TA injuries were excluded from the analysis, as they had a different causal relationship between the degree of TA damage and patient condition than in the in-car TA cases.

### Baseline characteristics

During the survey period, questionnaire responses and data on settlements were obtained from 619 patients. The mean age was 36.99 years, and the percentages of males and females were similar. Office job (26.25%) was the most common among respondents, followed by self-employment (15.54%), service/sales (13.93%), specialized (13.93%), and technician/engineering (12.32%). With respect to average monthly income, 2–4 million won accounted for 55.71%.

WAD Grade 2 (41.61%) was the most common, followed by Grade 1 (38.04%). The degree of damage to the car at the time of the TA was divided into six categories, and the most common response was Grade 3 (partially crushed, 46.61%), followed by Grade 4 (seriously crushed, 36.25%). With respect to the severity of pain immediately after the TA measured by NRS, the mean score was 6.01 points, while the largest group, at 45%, comprised those with NRS of 7–10 points. With respect to the severity of pain after CI settlement measured by NRS, the mean score was 3.48 points, while the largest group, at 58.39%, comprised those with NRS of 0–3 points. The mean duration between the TA and CI settlement was 92.33 days (Table 1).

**Table 1:** Baseline characteristics.

	Total		Male		Female	
	N	Mean±SD or %	N	Mean±SD or %	N	Mean±SD or %
**Age (years)**	560	36.99±9.62	266	36.81±8.88	294	37.15±10.25
19–30	127	22.68	54	20.3	73	24.83
30–40	261	46.61	136	51.13	125	42.52
40–50	101	18.04	46	17.29	55	18.71
50–60	52	9.29	22	8.27	30	10.2
60–70	19	3.39	8	3.01	11	3.74
**Job**						
Engineer	69	12.32	60	22.56	9	3.06
Office job	147	26.25	73	27.44	74	25.17
Self-employment	87	15.54	48	18.05	39	13.27
Service/sales	78	13.93	37	13.91	41	13.95
Research	15	2.68	7	2.63	8	2.72
Specialized	78	13.93	27	10.15	51	17.35
Housewife	54	9.64	0	0	54	18.37
Student	19	3.39	10	3.76	9	3.06
Others	13	2.32	4	1.5	9	3.06
**Income**^a^						
≤200	121	21.61	22	8.27	99	33.67
200–400	312	55.71	155	58.27	157	53.4
400≤	127	22.68	89	33.46	38	12.93
**WAD grade**						
0	9	1.61	5	1.88	4	1.36
1	213	38.04	105	39.47	108	36.73
2	233	41.61	114	42.86	119	40.48
3,4	105	18.74	42	15.79	63	21.43
**Degree of vehicle damage**^b^						
1,2	28	3.21	7	2.63	11	3.74
3	261	46.61	115	43.23	146	49.66
4	203	36.25	110	41.36	93	31.63
5,6	78	13.93	34	12.78	44	14.96
**Pain after TA (NRS)**		6.01±2.12		6.02±1.95		6.01±2.27
0–3	81	14.46	30	11.28	51	17.35
4–6	227	40.54	120	45.11	107	36.39
7–10	252	45.00	116	43.61	136	46.26
**Pain at CI settlement (NRS)**		3.48±1.997		3.28±2.02		3.65±1.96
0–3	327	58.39	166	62.41	161	54.76
4–6	185	33.04	77	28.95	108	36.73
7–10	48	8.57	23	8.65	25	8.5
**Duration to reach CI settlement**		92.33±151.38		97.7±165.81		87.49±137.13
≤1 week	66	11.79	38	14.29	28	9.52
1–4 weeks	173	30.89	80	30.08	93	31.63
1–3 months	176	31.43	85	31.95	91	30.95
3–6 months	68	12.14	24	9.02	44	14.97
6 months≤	77	13.75	39	14.66	38	12.93

SD, standard deviation; WAD, whiplash-associated disorder; TA, traffic accident; NRS, numeric rating scale; CI, car insurance.

^a^Monthly income (10,000 Korean won).

^b^Degree of vehicle damage, 1 (None), 2 (Scratches), 3 (Partially crushed), 4 (Seriously crushed), 5 (Broken in half), and 6 (Scrap).

### Association between degree of TA damage and pain and functional impairment after TA

The degree of damage to the car at the time of the TA was divided into four groups for analysis of association with pain and functional impairment after the TA. The results showed no significant differences in NRS immediately after the TA and in NDI, ODI, and EQ-5D-5L scores at admission, according to the degree of damage to the car (Table 2).

**Table 2:** Relationship of the degree of accident with post-accident pain and dysfunction.

Degree of vehicle damage^a^	Pain after TA (NRS)	NDI	ODI	EQ-5D-5L
1,2	5.50±2.73	45.56±9.69	43.44±11.68	0.77±0.08
3	5.97±2.08	52.38±13.45	49.31±13.74	0.74±0.12
4	6.10±2.05	53.67±12.25	49.99±12.76	0.73±0.12
5,6	6.062±2.33	53.10±12.08	49.56±13.11	0.74±0.11
**P-value**	0.662	0.071	0.2555	0.5883

The values were analyzed by the analysis of variance and are shown as mean ± standard deviation. TA, traffic accident; NRS, numeric rating scale; CI, car insurance; NDI, Neck Disability Index; ODI, Oswestry Disability Index; EQ-5D-5L, 5-level EuroQol 5-Dimension.

^a^Degree of vehicle damage, 1 (None), 2 (Scratches), 3 (Partially crushed), 4 (Seriously crushed), 5 (Broken in half), and 6 (Scrap).

### Associations of degree of TA damage and pain after TA with duration until reaching CI settlement

Associations of the degree of damage to the car at the time of TA, pain immediately after TA, and WAD grade with duration until reaching the CI settlement were analyzed. With respect to the degree of damage to the car, the CI settlement was reached sooner when the grade of damage was lower, except for grades 1 and 2. However, pain immediately after the TA and WAD grade did not show significant association with duration until reaching the CI settlement (Table 3).

**Table 3:** Relationship between degree of car accident/pain and the duration to reach CI settlement.

	Duration to reach CI settlement	P-value
Degree of vehicle damage^a^		0.003
1, 2	100.89±264.38	
3	74.38±124.39	
4	93.32±151.51	
5, 6	146.78±184.77	
Pain after TA (NRS)		0.461
0–3	81.53±182.24	
4–6	86.43±146.23	
7–10	100.82±144.91	
WAD grade		0.781
0	46±52.49	
1	89.28±166.74	
2	96.22±152.84	
3,4	93.10±117.20	

The values were analyzed by analysis of variance and are shown as mean ± standard deviation. CI, car insurance; TA, traffic accident; NRS, numeric rating scale; WAD, whiplash-associated disorder.

^a^Degree of vehicle damage, 1 (None), 2 (Scratches), 3 (Partially crushed), 4 (Seriously crushed), 5 (Broken in half), and 6 (Scrap).

### Analysis of factors influencing the duration and cost of treatment after CI settlement

Associations of pain at the time of the CI settlement with the duration and cost of treatment after the CI settlement were analyzed. The mean treatment cost was 24.47±113.4 (10,000 Korean won), and the mean duration of treatment was 4.14±13.24 weeks. The results showed that the cost was higher and the duration was greater when pain at the time of the CI settlement was more severe, and among females and in older age groups. Income level showed a significant association with treatment cost, but not with duration of treatment (Table 4). Regression analysis results also confirmed that the severity of pain at the time of the CI settlement had a significant influence on cost and duration of treatment after CI settlement (Tables 5 and 6) [23].

**Table 4:** Factors that affect the cost and duration of the treatment after car insurance settlement.

	Cost^a^		Duration^b^	
		P-value		P-value
**Total**	24.47±113.4		4.14±13.24	
**Pain after CI settlement (NRS)**				
0–3	11.02±49.74	0.0001	2.09±5.90	0.0001
4–6	24.37±62.22		5.92±18.39	
7–10	113.86±333.37		11.29±20.68	
**Sex**				
Male	18.87±131.12	0.265	2.73±12.06	0.016
Female	29.56±94.41		5.43±14.12	
**Age**				
19–30	8.94±25.70	0.001	2.73±6.60	0.033
30–40	19.68±69.17		3.92±13.36	
40–50	19.49±61.15		3.50±7.20	
50–60	75.12±289.03		7.40±24.75	
60–70	82.74±235.87		11.26±20.93	
**Income**^c^				
0–200	10.87±37.01	0.004	5.29±14.05	0.561
200–400	18.01±76.37		3.81±14.59	
400≤	53.35±200.70		3.87±7.88	
**Job**				
Engineer	8.81±23.69	0.450	2.97±14.50	0.195
Office job	20.23±63.80		3.64±12.47	
Self-employment	18.14±67.65		4.21±18.80	
Service/sales	40.29±229.58		3.71±7.87	
Research	47.67±135.24		8.20±14.98	
Specialized	21.01±80.67		3.01±6.55	
Housewife	51.81±152.91		8.98±17.47	
Student	10.53±31.53		1.05±3.22	
Others	4.92±7.69		4.85±8.96	

The values are shown as mean ± standard deviation. CI, car insurance; NRS, numeric rating scale.

^a^ Cost unit was 10,000 Korean won.

^b^ Duration unit was one week.

^c^ Monthly income (10,000 Korean won).

**Table 5:** Results of regression analysis showing factors that affect the cost of treatment after car insurance settlement.

	Model 1 Parameter Estimate	Standard Error	Pr>|t|	Model 2 Parameter Estimate	Standard Error	Pr>|t|	Model 3 Parameter Estimate	Standard Error	Pr>|t|
**Pain after CI settlement (NRS)**									
	0.23	0.03	< .0001	0.21	0.03	< .0001	0.21	0.03	< .0001
**Sex**									
Male									
Female				0.49	0.14	0.0004	0.74	0.15	< .0001
**Age**									
				0.02	0.01	0.0105	0.01	0.01	0.0772
**Income**									
0≤, ≤200									
200≤, ≤400							0.60	0.22	0.0052
400≤							1.29	0.25	< .0001
**Job**									
Engineer									
Office job							-0.02	0.24	0.9188
Self-employment							-0.32	0.26	0.2145
Service/Sales							-0.08	0.27	0.7541
Research							-0.38	0,46	0.4088
Specialized							-0.44	0.27	0.1124
Housewife							0.34	0.35	0.3297
Student							0.23	0.45	0.6136
Other							0.09	0.50	0.8539
**R-squared**			0.071308			0.102574			0.151935
P-value			< .0001			< .0001			< .0001

CI: Car Insurance; NRS: numeric rating scale.

**Table 6:** Results of regression analysis showing factors that affect the duration of treatment after car insurance settlement.

	Model 1 Parameter Estimate	Standard Error	Pr>|t|	Model 2 Parameter Estimate	Standard Error	Pr>|t|	Model 3 Parameter Estimate	Standard Error	Pr>|t|
**Pain after CI settlement (NRS)**									
	0.17	0.02	< .0001	0.16	0.02	< .0001	0.16	0.02	< .0001
**Sex**									
Male									
Female				0.39	0.09	< .0001	0.43	0.10	< .0001
**Age**									
				0.01	0.00	0.0046	0.01	0.00	0.532
**Income**									
0≤, ≤200									
200≤, ≤400							0.10	0.14	0.482
400≤							0.34	0.16	0.0387
**Job**									
Engineer									
Office job							-0.03	0.15	0.8598
Self-employment							-0.16	0.17	0.3591
Service/Sales							-0.05	0.18	0.76
Research							0.04	0,30	0.8798
Specialized							-0.22	0.18	0.2169
Housewife							0.20	0.23	0.3889
Student							-0.14	0.29	0.6433
Other							0.23	0.32	0.4805
**R-squared**			0.096314			0.13981			0.155932
P-value			< .0001			< .0001			< .0001

CI: Car Insurance; NRS: numeric rating scale.

### Satisfaction in symptom improvement at the time of CI settlement and reason(s) for CI settlement

**Fig 1:**
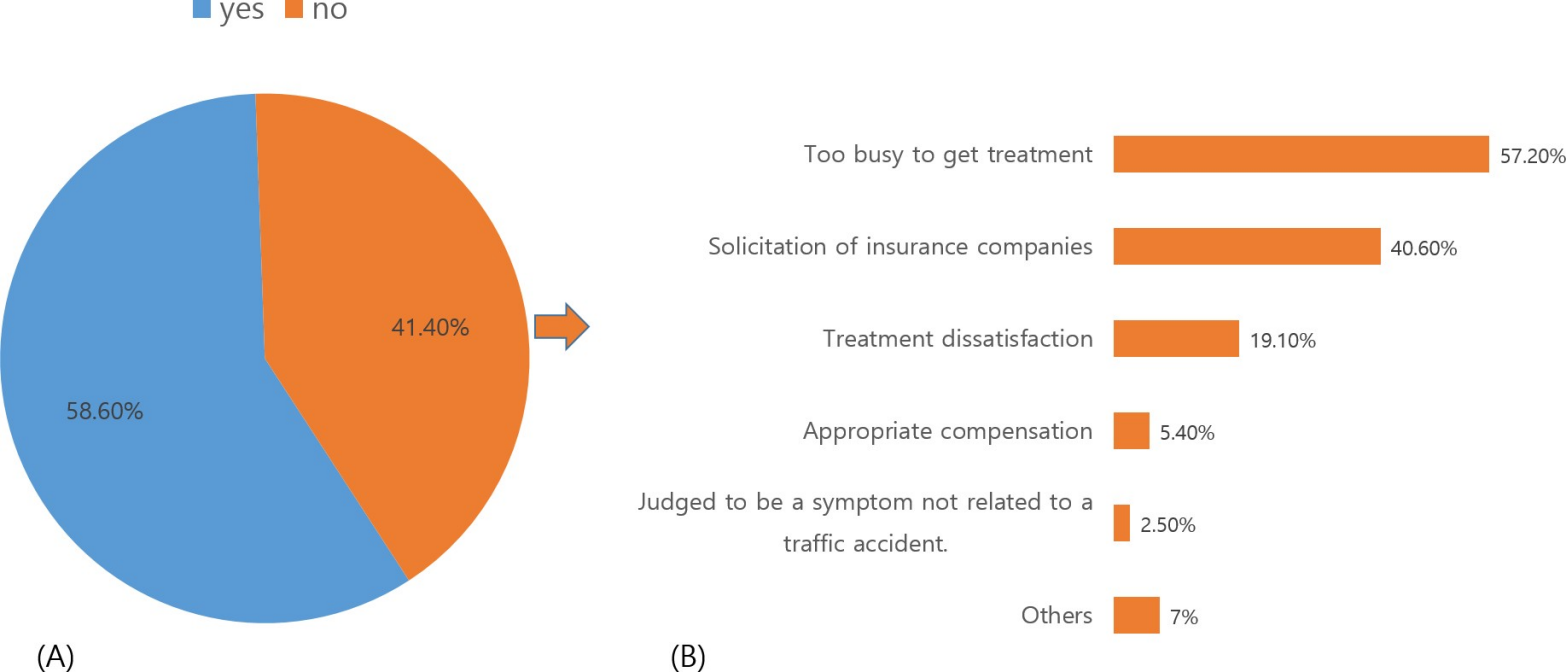
Satisfaction at the time of the settlement and the reason for settlement, despite dissatisfaction. (A) Improvement satisfaction ratio (n = 619); (B) Reasons for settlement despite no improvement in health (n = 256).

At the time of the CI settlement, 58.6% of patients were satisfied with the settlement, whereas 41.4% were not satisfied. Considering the reasons these patients settled despite not being satisfied, “being too busy to receive treatment” (57.2%) was the most common, while “recommendation to settle by the insurance agent” (40.6%) accounted for a high percentage as well. Other reasons included “dissatisfaction with the treatment” (19.1%) and “adequate settlement amount” (5.4%) (Fig 1).

### Satisfaction in CI system and KM treatment

**Fig 2:**
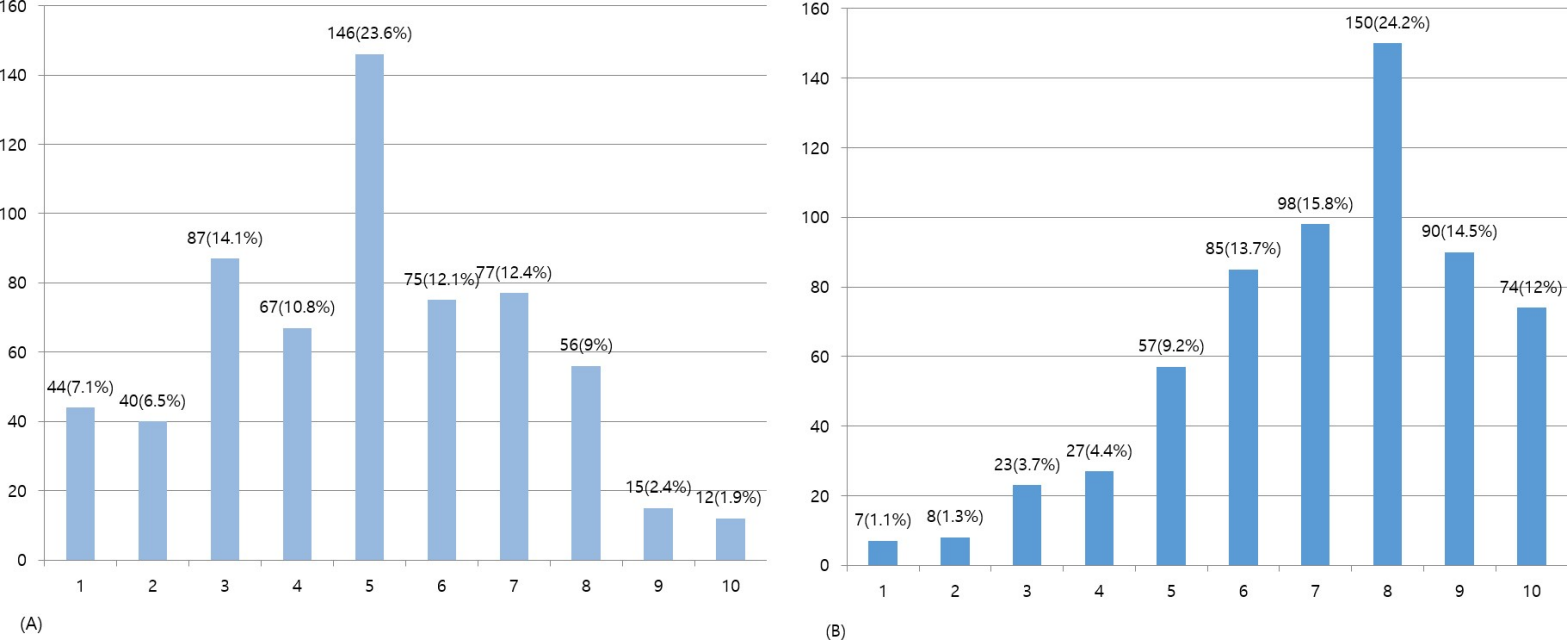
Satisfaction score of car insurance system and Korean medicine treatment for traffic accidents (n = 619). (A) Satisfaction score of the car insurance system; (B) Satisfaction with Korean medicine treatment for traffic accidents.

**Fig 3:**
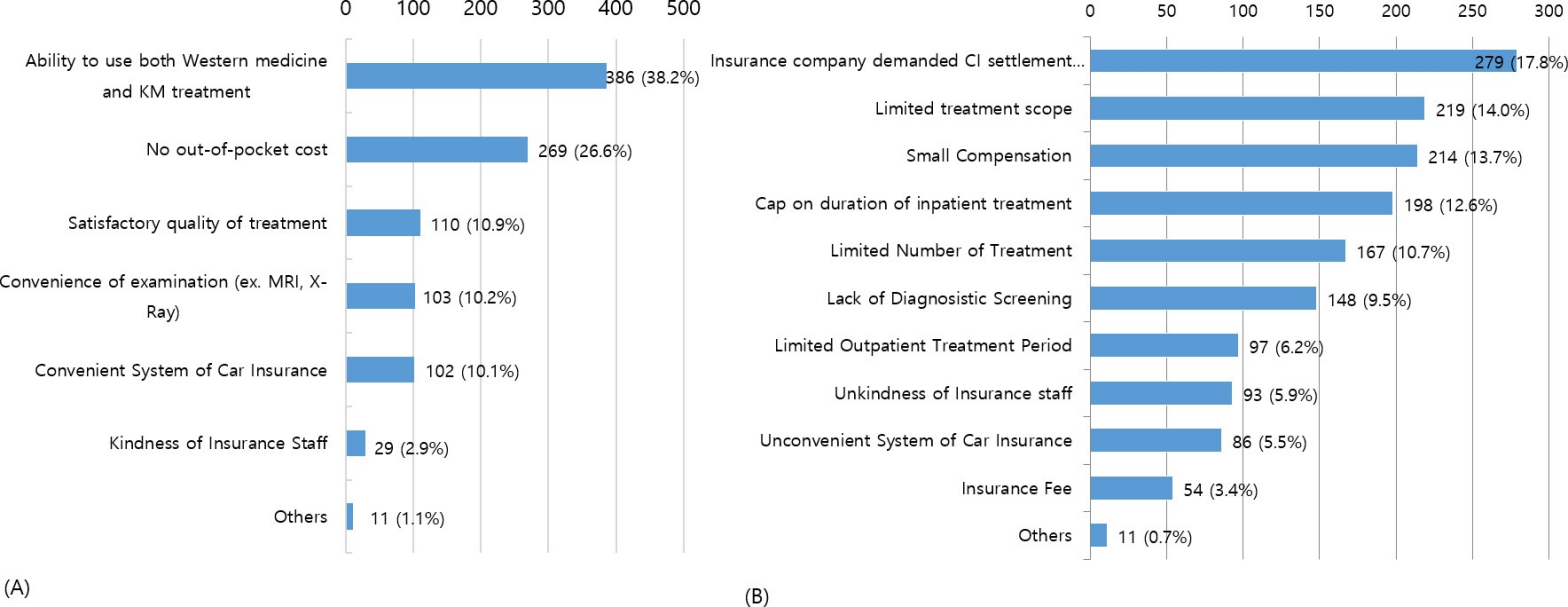
Satisfactory and unsatisfactory factors of car insurance system. (A) Satisfactory factors of the car insurance system (n = 619, duplicate responses allowed); (B) Unsatisfactory factors of the car insurance system (n = 619, duplicate responses allowed).

The mean score for satisfaction in the CI system was 4.97 points, and the mean score for satisfaction in KM treatment for TA injuries was 7.16 points (Fig 2). With respect to the most satisfactory aspects in the current CI system, the “ability to use both Western medicine and KM treatment” (38.2%) was the most common response, followed by “no out-of-pocket cost” (26.6%) and “satisfactory quality of treatment” (10.9%). In contrast, the unsatisfactory aspect of the current CI system was that the “insurance company demanded CI settlement despite wanting additional treatment for still being hurt” (17.8%), followed by “limited treatment scope” (14.0%) and “small compensation” (13.7%) (Fig 3). The most satisfactory treatment among various forms of KM treatment was Chuna (31.8%), followed by pharmaco-acupuncture (24.0%), acupuncture (22.6%), herbal therapy (13.0%), cupping (7.6%), and others (1.0%).

## Discussion

Analysis showed that the association between the degree of TA damage and severity of pain immediately after the TA was not always proportional. According to an existing study [11], when indicators or functional impairment such as NDI, ODI, and EQ-5D-5L, were analyzed together, these indicators were not always proportional to the degree of TA damage. Such results indicate that it is not easy to ascertain the severity of pain based on the degree of damage to the car alone. This suggests that because the acceleration/deceleration mechanism causes whiplash, the degree of acceleration/deceleration involved has a larger influence on pain than the degree of damage the car sustained. The association between the severity of injury and impact and velocity has been controversial. Freeman et al. [24] investigated injury rates at amusement parks, which do not appear to be dangerous on the outside, and found that injuries could occur even at very low velocity. The findings in the present study also supported these results. Moreover, a whiplash is often undetectable by diagnostic tests, including X-ray and magnetic resonance imaging [25]; therefore, a more thorough assessment may be needed of the severity of pain, medical history, and functions when assessing a patient’s pain.

A related study reported that even after relatively minor TAs, symptoms such as limitations in range of motion, cervical muscle spasms, tingling, visual and auditory disorders, dizziness, and nausea occurred in addition to injury to the anterior longitudinal ligament of the cervical spine, fractures of the vertebral end plate, disc injury, and posterior longitudinal ligament injury in magnetic resonance imaging findings [23]. Another study reported that even if the clinical symptoms disappeared after the whiplash injury, muscle strength and endurance in the cervical spine were not restored to normal for a long time, and, in particular, range of motion limitation in extension compared to that in flexion was more severe up to one year immediately after the accident [26].

Analysis of correlations with factors influencing the duration until reaching CI settlement showed a positive correlation with the degree of damage to the car, but there was no significant correlation with WAD grade or pain immediately after TA. Therefore, there is high likelihood of a perception that treatment is unnecessary in cases with little damage to the car because these are classified as mild accidents, regardless of the severity of the pain or status of the patient. Therefore, there is a tendency to decide on a CI settlement with a determination based on the degree of damage to the car. The results indicated that a CI settlement was reached quickly without patients receiving sufficient treatment in 41.4% of cases. While some cases were due to the patient being too busy to receive treatment, others involved continued recommendations from the insurance company to settle and dissatisfaction in treatment options that could be received through CI. Generally, the start and end of treatment and treatment modalities are considered aspects that need to be decided by medical professionals. However, having an insurance agent, who is a non-medical professional, recommend the discontinuation of treatment could be a factor that interferes with the patient receiving sufficient treatment.

The aspects of the current CI system that satisfied most respondents according to the questionnaire survey were not having out-of-pocket costs and the ability to use both Western medicine and KM treatment. Under the dualistic healthcare system of South Korea, KM treatment is often used for pain caused by a TA. In the questionnaire survey, 84.2% of the respondents had received KM treatment only or switched to KM treatment from Western medicine treatment for a TA injury. The mean score for satisfaction in KM treatment for a TA injury (scale of 0–10) was 7.16 points. Research indicates that such high satisfaction in KM treatment is based on the efficacy of acupuncture or manual therapy for musculoskeletal disorders [14, 27–29]. KM treatment is often used for pain from musculoskeletal disorders caused by TAs in South Korea, and during 2014–2018, the annual average number of cases claimed for CI review involving a KM hospital and a clinic increased by 23.4% and 11.3%, respectively [30].

The most satisfactory treatment modality was Chuna therapy (32%), followed by pharmaco-acupuncture (24%) and acupuncture (23%). Chuna therapy is a form of manual therapy performed personally by the KM practitioner by using his or her hands or other parts of the body and with instruments such as a Chuna table [31]. It is effective for reducing pain in musculoskeletal disorders [28, 32–35]. Manual therapy is used most often for musculoskeletal pain, along with drug and exercise therapy [36]. Miao et al. [37] reported that patients with musculoskeletal pain preferred manual therapy over exercise therapy and that manual therapy showed greater short-term efficacy. Pharmaco-acupuncture is a newer acupuncture technique that combines acupuncture and herbal therapy, and many studies have identified it efficacy in controlling pain [29, 38, 39]. Mechanical and chemical stimulation could be sought simultaneously during the procedure [40], and it also has an excellent safety record [41], resulting in its use as a treatment modality for various musculoskeletal disorders. In addition, previous studies have reported on the effectiveness of herbal therapy in treating neck pain [42], traumatic brain injury [43] and pain caused by blood stasis.

The goal of CI is timely recovery for patients with a TA injury, whereas the frequency of treatments is concentrated in the early stage. Therefore, if early treatment opportunities are missed, it might result in limiting the utilization of healthcare services. Previous studies have reported that more than half of patients with a TA injury require three to six months for recovery and one out of three or four patients have persistent pain that lasts up to two years after the accident [44–46]. In addition, it has been reported that the most common symptoms six weeks after motor vehicle collisions with mild traumatic brain injury were sleep disorders (65%), fatigue (59%), neck pain (50%), headache (39%), dizziness (39%), and low back pain (35%), and more than half of the patients complained of more than three symptoms after a year post-collision, while 17% of them showed more than eight symptoms [47]. Therefore, it is important to prevent such pain from becoming chronic pain. Sterling et al. [48] reported that developing chronic pain is highly likely if there is no significant improvement in symptoms or appropriate treatment is not received within the first three months after the accident. Berglund et al. [49] reported that patients with acute neck pain at the time of a TA are three times more likely to develop chronic pain than regular patients with neck pain. These studies indicate the importance of receiving appropriate treatment in the early stage of TA injury. Frequently, however, people may reach a CI settlement before their complete recovery or spend time and money treating lingering pain after a CI settlement due to inadequate coverage by the CI system. Such expenditures would be borne by the National Health Insurance, causing waste. Accordingly, there is a need for a balanced approach to alleviate the financial burden on the National Health Insurance and achieve sufficient recovery in patients with TA injury. Considering that previous studies generally reported that the severity of symptoms lasted for up to six weeks [50], the course of symptom recovery continued over three to six months, and there was a need for more than one year of treatment [5, 50, 51], in combination with the results of this study that investigated the time to reach CI settlement, sufficient treatment would need to be provided for at least three months on average for patients in acute to subacute phase.

Unlike previous studies, this was a large-scale study that obtained responses from 619 patients. Accordingly, the study has some notable strengths. The study directly engaged the patients to investigate the circumstances and severity of pain at the time of accident and their satisfaction in the treatment received. Data regarding severity of pain and functional scales at the time of admission were also compiled in carrying out the analysis. Another noteworthy point is that the study highlighted the circumstances at the time of the CI settlement by examining the duration from the accident to the CI settlement and situations thereafter. Existing study reports have often used limited KM treatment modalities, such as just acupuncture [52–54] or manual therapy [3, 55], but the present study investigated alleviation of pain and satisfaction in treatment by applying not only acupuncture or cupping, but integrative KM treatment that included Chuna, pharmaco-acupuncture, and herbal therapy.

Nonetheless, the present study also has some limitations. Due to the nature of a questionnaire survey, recall bias may have occurred due to dependency on memory. In the open-ended questions, each participant provided a variety of different responses, which presented difficulties in summarizing the responses. Moreover, because patient data at the time of admission were compiled retrospectively, some data may have contained errors and omissions due to computer or human errors. TAs characteristically occur more often in those aged 30–49 years who are more socially active. However, the response rate was relatively lower among those aged ≥50 years who are frequently less familiar with the use of electronic devices such as those for the online survey, meaning the study was conducted with a relatively young study population [2]. Furthermore, because the participants in the present study received combination KM therapy consisting of different treatment modalities, the study could not analyze which treatment modality was the most effective in alleviating pain. To address these issues, future questionnaire surveys should avoid open-ended questions as much as possible and include mostly multiple-choice questions, while the data collection period should be longer to allow text messages to encourage and promote participation in the survey. Moreover, various survey formats, including telephonic and face-to-face interviews and mail surveys, should be used in combination to obtain responses from broader age groups.

The comprehensive consideration of analysis results indicates that greater severity of pain at the time of a CI settlement leads to greater treatment cost and duration with health insurance expenses being paid as out-of-pocket cost. Therefore, it is necessary to have social discussions on the proper balance between the duration for reaching a CI settlement and coverage for treatment. Specifically, in consideration of the study finding that no significant association was found between the degree of pain in the early stage of a TA and the degree of vehicle damage, it is necessary to develop a policy for sufficient assurance of patients’ rights to receive treatment even after a minor TA. In addition, as treatment using out-of-pocket cost or with National Health Insurance continues for residual pain after the settlement, supplementation of the policy to reduce confusion between the purposes of the National Health Insurance and CI is needed. Taking into account the high level of patients’ satisfaction with Chuna therapy, pharmaco-acupuncture, and acupuncture, and many patients expressing dissatisfaction with insufficient treatment before reaching the settlement, it is thought to be important to prevent the prolonged sequelae of patients through more careful and probing deliberation on a policy for sufficient treatment of patients in acute to subacute phase.

## Conclusions

The findings of this study demonstrate that there is no significant association between the degree of damage to the car in a TA and pain after a TA. However, the duration of reaching a CI settlement was more significantly associated with the degree of damage to the car than the severity of pain. Greater severity of pain at the time of the CI settlement was associated with greater cost and longer time spent in treatment after the CI settlement. Furthermore, KM treatment showed generally high scores from treatment and satisfaction aspects with respect to treatment for a TA injury. Accordingly, such findings need to be considered in finding an appropriate balance between the duration of treatment and the CI settlement when establishing future policies on CI and National Health Insurance.
